# Respiratory Virus Detection and Sequencing from SARS-CoV-2–Negative Rapid Antigen Tests

**DOI:** 10.3201/eid3113.241191

**Published:** 2025-05

**Authors:** Emmanuela Jules, Charlie Decker, Brianna Jeanne Bixler, Alaa Ahmed, Zijing (Carol) Zhou, Itika Arora, Henok Tafesse, Hannah Dakanay, Andrei Bombin, Ethan Wang, Jessica Ingersoll, Kathy Bifulco, Jennifer K. Frediani, Richard Parsons, Julie Sullivan, Morgan Greenleaf, Jesse J. Waggoner, Greg S. Martin, Wilbur A. Lam, Anne Piantadosi

**Affiliations:** Emory University School of Medicine, Atlanta, Georgia, USA (E. Jules, C. Decker, B.J. Bixler, A. Ahmed, Z.(C.) Zhou, I. Arora, H. Tafesse, H. Dakanay, A. Bombin, E. Wang, J. Ingersoll, J. Sullivan, J.J. Waggoner, W.A. Lam, A. Piantadosi); Emory Integrated Genomics Core, Winship Cancer Institute of Emory University, Atlanta (A. Ahmed); Nell Hodgson Woodruff School of Nursing, Emory University, Atlanta (K. Bifulco, J.K. Frediani, R. Parsons); The Atlanta Center for Microsystems Engineered Point-of-Care Technologies, Atlanta (J.K. Frediani, R. Parsons, J. Sullivan, M. Greenleaf); Georgia Clinical and Translational Science Alliance, Atlanta (M. Greenleaf); Emory University Division of Pulmonary, Allergy, Critical Care Medicine and Sleep Medicine, Atlanta (G.S. Martin); Wallace H. Coulter Department of Biomedical Engineering, Georgia Institute of Technology and Emory University, Atlanta (W.A. Lam); Aflac Cancer and Blood Disorders Center of Children's Healthcare of Atlanta, Atlanta (W.A. Lam)

**Keywords:** molecular epidemiology, respiratory tract infections, public health surveillance, SARS-CoV-2, COVID-19, coronavirus disease, severe acute respiratory syndrome coronavirus 2, viruses, respiratory infections, zoonoses

## Abstract

Genomic epidemiology offers insight into the transmission and evolution of respiratory viruses. We used metagenomic sequencing from negative SARS-CoV-2 rapid antigen tests to identify a wide range of respiratory viruses and generate full genome sequences. This process offers a streamlined mechanism for broad respiratory virus genomic surveillance.

The COVID-19 pandemic highlighted the importance of genomic epidemiology in understanding virus transmission and evolution, informing essential countermeasures from nonpharmaceutical interventions to vaccines. Massive global efforts in SARS-CoV-2 genomic surveillance were made possible by widespread diagnostic testing and the growth of new infrastructure and methods for sequencing and analysis (*1*). Most genomic surveillance pipelines in the United States obtained residual SARS-CoV-2–positive samples from clinical, public health, and commercial laboratories. That strategy was effective during the pandemic but difficult to maintain with the rise of at-home rapid antigen tests (*2*,*3*). As traditional sample sources declined, our group and others demonstrated that residual samples from rapid antigen tests could be used to generate and analyze full SARS-CoV-2 sequences for genomic surveillance (*4*–*6*).

In this study, we build upon that work by identifying, sequencing, and analyzing other respiratory viruses using residual swab samples from negative BinaxNOW COVID-19 antigen tests (Abbott, https://www.abbott.com). This multivirus approach is key because SARS-CoV-2 has transitioned to an endemic virus whose symptoms resemble those of other respiratory viruses (*7*). Thus, there is both a need for broad testing and an opportunity to expand genomic surveillance for respiratory viruses using self-collected samples.

## Methods

**Figure 1:**
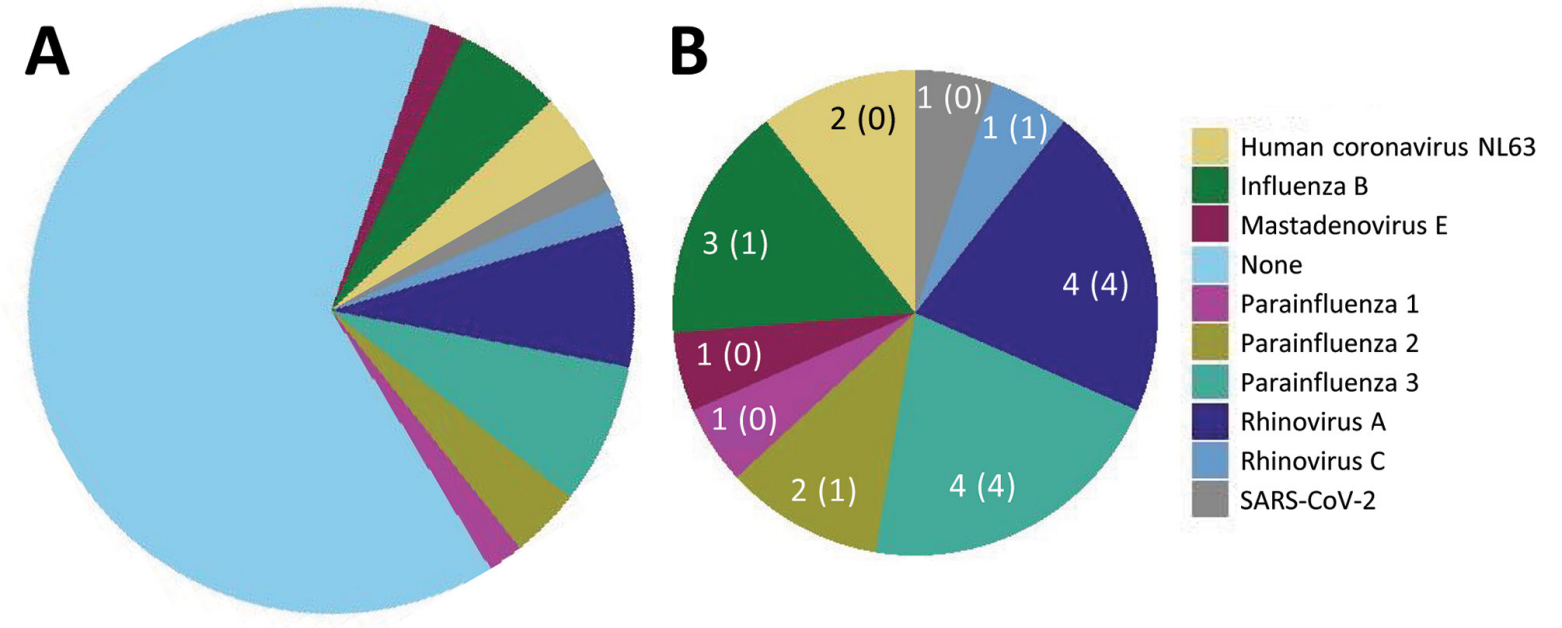
Frequency of human pathogenic respiratory viruses found in 53 residual samples from SARS-CoV-2–negative BinaxNOW tests (Abbott, https://www.abbott.com) in study of respiratory virus detection and sequencing from negative rapid antigen tests. Pie charts indicate the number of samples positive for each virus among all samples (A) and among the 18 positive samples (B). Numbers indicate the number of samples with a virus identified, followed in parentheses by the number of samples with a >90% complete genome sequence assembled.

In brief, participants were enrolled in a parent study evaluating novel viral diagnostic tests through the RADx program at the Atlanta Center for Microsystems Engineered Point-of-Care Technologies (Atlanta, GA, USA). The study protocol was approved by the Emory University Institutional Review Board and the Grady Health Research Oversight Committee (both in Atlanta). We performed RNA metagenomic sequencing as described (*8*), obtaining a median of 5.8 million reads per sample (Appendix 1; Appendix 2). We used a 3-step bioinformatic approach to detect viruses (Appendix 1 Figure 1) using KrakenUniq (https://github.com/fbreitwieser/krakenuniq), blastn (https://blast.ncbi.nlm.nih.gov/Blast.cgi?PROGRAM=blastn&PAGE_TYPE=BlastSearch&LINK_LOC=blasthome), and reference mapping (https://github.com/briannajeanne/metagen/tree/main). Our final criterion required coverage of >10% of the viral genome, or reads mapping to >3 nonoverlapping regions of the viral genome with >80% identity, similar to clinical diagnostic criteria that have previously been used for metagenomic sequencing (*9*).

## Results

We collected negative BinaxNOW test samples from 53 persons during April–August 2023 (Appendix 1 Table), a period during which 68% of the BinaxNOW tests in the parent study were negative. All persons were symptomatic at the time of testing (Table), and the median interval between symptom onset and testing was 2 (range 0–9) days. Reverse transcription PCR (RT-PCR) was positive for influenza B in 3 samples and negative for influenza A, respiratory syncytial virus, and SARS-CoV-2 in all samples (Appendix 2).

**Table:** Participant symptoms in study of respiratory virus detection and sequencing from SARS-CoV-2–negative rapid antigen tests*

Symptom	Total participants, N = 53	Participants with a virus detected, n = 18†	Participants with no virus detected, n = 35
Upper respiratory	47 (88.7)	17 (94.4)	30 (85.7)
Congestion/runny nose	37 (69.8)	15 (83.3)	22 (62.9)
Sore throat	33 (62.3)	14 (77.8)	19 (54.3)
Loss of sense of taste or smell	7 (13.2)	2 (11.1)	5 (14.3)
Lower respiratory	43 (81.1)	15 (83.3)	28 (80.0)
Cough	39 (73.6)	15 (83.3)	24 (68.6)
Shortness of breath	23 (43.4)	6 (33.3)	17 (48.6)
Gastrointestinal	15 (28.3)	6 (33.3)	9 (25.7)
Vomiting	4 (7.6)	3 (16.7)	1 (2.9)
Nausea	11 (20.8)	2 (11.1)	9 (25.7)
Diarrhea	2 (3.8)	1 (5.6)	1 (2.9)
Abdominal pain	7 (13.2)	2 (11.1)	5 (14.3)
Systemic	35 (66.0)	13 (72.2)	22 (62.9)
Fever, temperature >100.4°F	18 (34.0)	7 (38.9)	11 (31.4)
Chills	24 (45.3)	11 (61.1)	13 (37.1)
Fatigue	27 (50.9)	11 (61.1)	16 (45.71)
Other	41 (77.4)	16 (88.9)	25 (71.4)
Headache	30 (56.6)	13 (72.2)	17 (48.6)
Joint pain	14 (26.4)	3 (16.7)	11 (31.4)
Muscle pain	31 (58.5)	10 (55.6)	21 (60.0)

*Values are no. (%) participants reporting each symptom at the time of testing. For symptom categories, the number of participants with >1 symptom in that category is reported. †Includes 1 person in whom SARS-CoV-2 was detected at a low level and 17 persons in whom an alternative human pathogenic respiratory virus was detected.

Metagenomic sequencing identified a low level of SARS-CoV-2 in 1 sample and a different pathogenic human respiratory virus in 17 (33%) of the other 52 samples (Appendix 2). We detected parainfluenza viruses (n = 7), rhinoviruses (n = 5), influenza B (n = 3), seasonal coronaviruses (n = 2), and adenovirus (n = 1) (Figure 1). In 1 sample, we detected both influenza B and parainfluenza 2. In another sample positive for influenza B by RT-PCR, metagenomic sequencing did not identify influenza but identified human mastadenovirus E. Thus, excluding SARS-CoV-2, we detected a total of 18 viruses across 17 samples.

The duration of time between sample collection and nucleic acid extraction was similar for samples with a virus detected (median 6 [range 4–12] days) and samples with no virus detected (median 7 [range 5–19] days). RT-PCR for RNase P was positive in all samples tested, and the percentage of human reads was similar between samples with and without viruses detected (p = 0.07 by Mann-Whitney U test) (Appendix 2). We saw no difference in the total number of reads obtained for samples with and without viruses detected (p = 0.29 by Mann-Whitney U test).

We compared potential differences in symptoms between persons in whom a virus was detected and those in whom no virus was detected and observed the following disparities: congestion (83% vs. 63%), sore throat (78% vs. 54%), chills (61% vs. 37%), and headache (72% vs. 49%) (Table). However, none of those differences were statistically significant. The time between symptom onset and testing was similar between persons with a virus detected (median 2 [range 0–9] days) and those without a virus detected (median 2 [range 0–6] days).

Of the 18 viruses detected, we generated full viral genome sequences from 11 (61%) with >90% coverage and 71- to 24,000-fold depth (Appendix 2). Those 11 sequences consisted of parainfluenza 3 (4/4 samples), parainfluenza 2 (1/2), rhinovirus (5/5), and influenza B (1/3).

**Figure 2:**
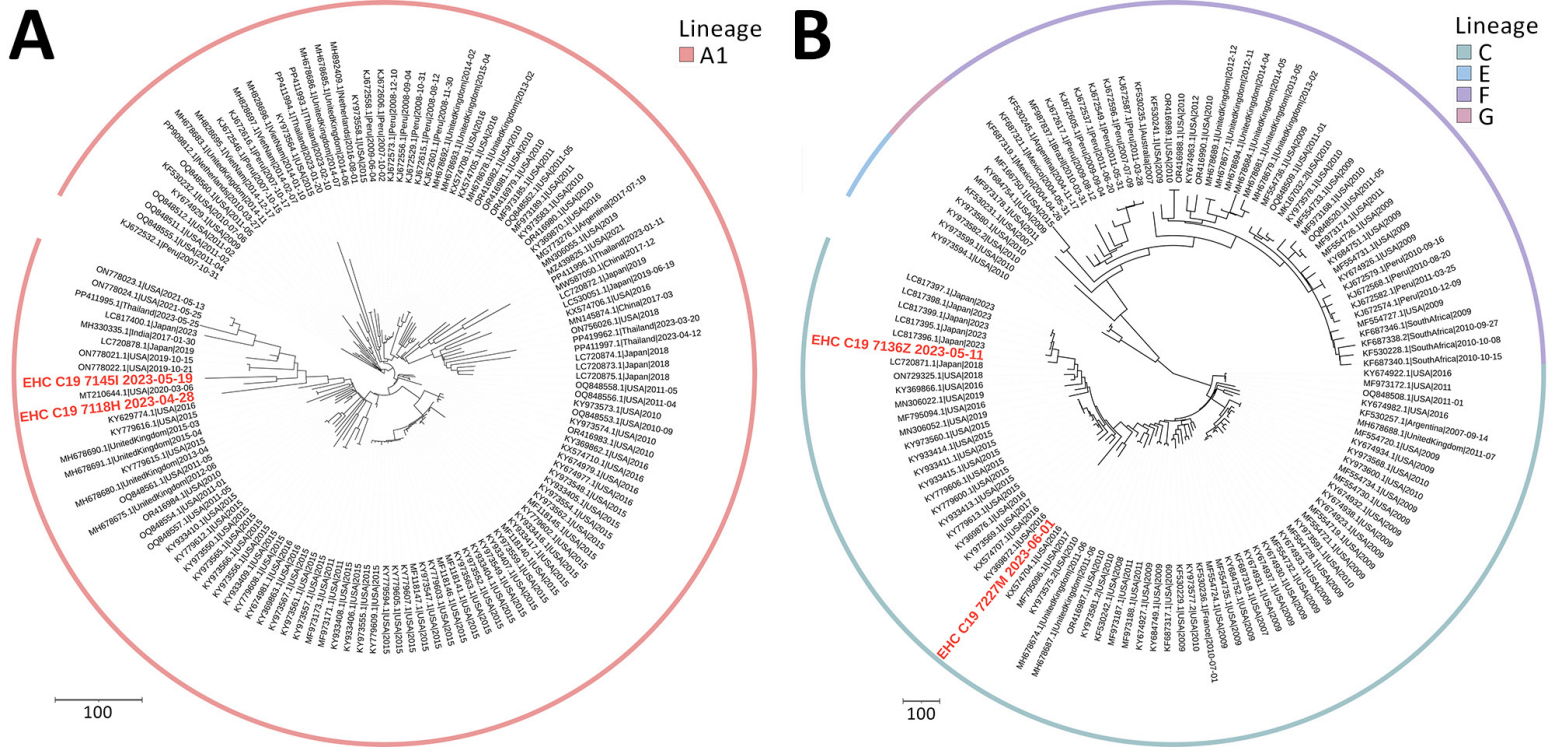
Phylogenetic analysis of parainfluenza 3 virus sequences in study of respiratory virus detection and sequencing from SARS-CoV-2–negative rapid antigen tests. The names of sequences obtained in this study are bold and in red, and reference sequences are in black. The outer ring indicates virus lineage. A) Representative sequences from lineage A1; B) representative sequences from lineages C, E, F, and G. Each tree is a maximized parsimony subtree using downsampled data from the full analysis in Appendix 1 Figure 2), for ease of visualization. GenBank accession numbers are provided for reference sequences. Scale bars indicate number of substitutions per site.

We performed phylogenetic analysis of parainfluenza 3 as a proof-of-concept for genomic epidemiology studies and found substantial diversity. Using the lineage classification system described in Lee et al. (*10*), 2 of our sequences clustered with lineage A1 sequences from 2019–2023 (Figure 2, panel A), another clustered with lineage C sequences from Japan in 2023, and the fourth with lineage C sequences from the United States collected during 2015–2017 (Figure 2, panel B), all with high bootstrap support (Appendix 1 Figure 2). Of note, only ≈450 complete parainfluenza 3 virus sequences are available; the data from our small study represent nearly 1% of this number, underscoring the opportunity to easily expand genomic surveillance using this approach.

**Figure 3:**
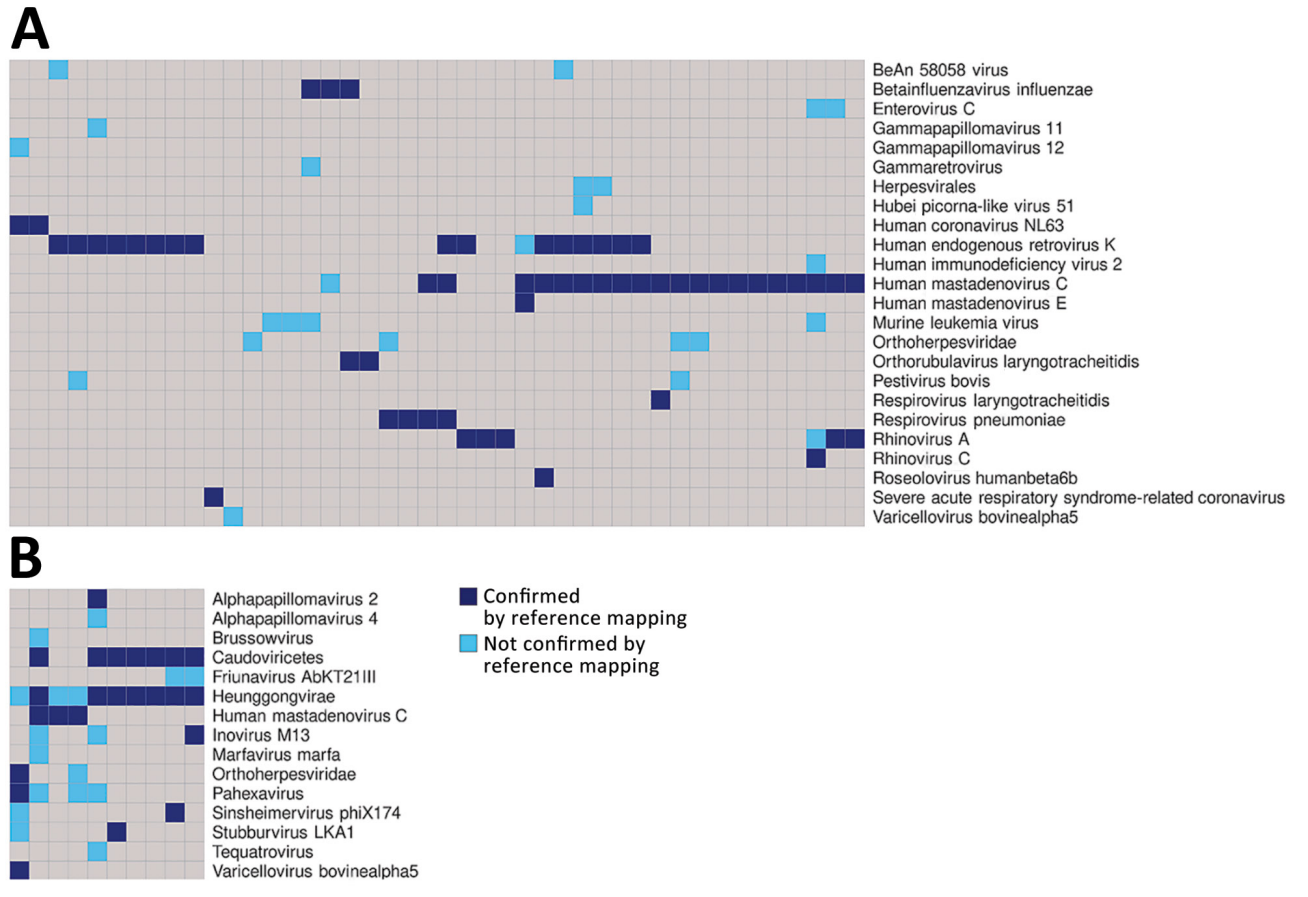
Plot of the viral taxa (rows) that were detected in each sample (columns) in study of respiratory virus detection and sequencing from SARS-CoV-2–negative rapid antigen tests. A) Results from samples used in this study; B) results from negative controls. Dark blue boxes indicate viruses that were detected by both KrakenUniq (https://github.com/fbreitwieser/krakenuniq) and blastn (https://blast.ncbi.nlm.nih.gov/Blast.cgi?PROGRAM=blastn&PAGE_TYPE=BlastSearch&LINK_LOC=blasthome) and were confirmed by reference mapping (covering >3 distinct regions or 10% of the reference virus genome). Light blue boxes indicate viruses that were detected by both KrakenUniq and blastn but not confirmed by reference mapping. This figure only includes results for which >1 read mapped to a reference genome sequence. Further detail including sample identifiers is shown in Appendix 1 Figures 3,4.

In addition to human pathogenic respiratory viruses, we detected >100 viruses of no clinical significance, including bacteriophages and plant viruses, many of which were also detected in our negative controls (Figure 3; Appendix 1 Figures 3, 4). Similarly, we found mastadenovirus C in about one third of all samples and negative controls, all with low genome coverage (Appendix 3). Those findings are all consistent with environmental or reagent contaminants. Herpesviruses were reported in many samples by KrakenUniq and blastn but generally were not confirmed by reference mapping. One adult participant had confirmed detection of human herpesvirus 6, which, given the participant’s age, more likely reflects latent virus than acute infection. Overall, 1,367 viral taxa were identified by KrakenUniq, only 254 (18.6%) were confirmed by blastn, and only 137 (53.9% [10% of total]) met our criteria for detection (Appendix 3), highlighting the importance of confirmatory steps in metagenomic analysis.

## Discussion

Our study demonstrates that RNA metagenomic sequencing of residual swab samples from negative BinaxNOW COVID-19 tests can be used to detect a broad range of respiratory viruses, including rhinoviruses, parainfluenza viruses, influenza B, seasonal coronaviruses, and adenovirus. All of those viruses have overlapping symptoms, both with one another and with SARS-CoV-2, underscoring the need for multivirus testing approaches. Although our study was not designed for clinical diagnosis, metagenomic sequencing is increasingly used clinically, and our results illustrate the need for rigorous analysis techniques and careful interpretation.

Of note, only 33% of samples had a human pathogenic respiratory virus. This finding is similar to that of our previous study, in which alternative respiratory viruses were detected in only 40% of SARS-CoV-2–negative persons using residual clinical samples early in the pandemic (*8*). Possible explanations include persons with a noninfectious syndrome, a bacterial or other nonviral infection, or a virus present at a low level. Some persons could also have been infected with a DNA virus not optimally captured by RNA sequencing. However, we detected adenovirus, the most prevalent respiratory DNA virus. Among common RNA viruses, we did not detect influenza A or respiratory syncytial virus, which we attribute to the winter-predominant seasonality of these viruses, whereas our samples were collected in spring and summer.

Of note, of the 18 viruses detected, we were able to generate full viral genome sequences from 11 (61%) using moderate sequencing depths. Thus, the single laboratory technique of metagenomic sequencing can not only identify diverse respiratory viruses but also contribute to their genomic surveillance. The surprisingly high depth of genome coverage achieved for many sequences indicates that throughput and cost can be improved by reducing total sequencing reads from each sample in future studies.

By combining metagenomic sequencing with the use of residual antigen test samples, we demonstrate a mechanism for convenient and broad respiratory virus surveillance. Our study used BinaxNOW tests, which conveniently preserve the used swab within the kit cassette; future work is needed to evaluate this approach using rapid antigen test strips themselves, as previously demonstrated for SARS-CoV-2 sequencing (*5*). In addition, future studies would benefit from a regulatory framework in which, after rigorous analysis and careful interpretation, clinically significant results can be returned to study participants, who are likely curious about the presence of other respiratory viruses when rapid antigen testing is negative for COVID-19. In conclusion, our study illustrates that residual samples from self-collected antigen tests can be a powerful sample source for investigating the genomic epidemiology of a broad range of respiratory viruses, building upon the strong foundations for viral surveillance established during the COVID-19 pandemic.

Appendix 1 Additional information about respiratory virus detection and sequencing from SARS-CoV-2–negative rapid antigen tests

Appendix 2 Additional data from study of respiratory virus detection and sequencing from SARS-CoV-2–negative rapid antigen tests

Appendix 3 Additional data used in study of respiratory virus detection and sequencing from SARS-CoV-2–negative rapid antigen tests
