# Supplementary material for: Respiratory Virus Detection and Sequencing from SARS-CoV-2–Negative Rapid Antigen Tests
Source: Emerg Infect Dis. 2025 May;31(Suppl 1):S39–44. doi: 10.3201/eid3113.241191 (PMC12078552; doi:10.3201/eid3113.241191)
Supplement: Appendix 1 — Additional information about respiratory virus detection and sequencing from SARS-CoV-2–negative rapid antigen tests [file 24-1191-Techapp-s1.pdf]

Article DOI: https://doi.org/10.3201/eid3113.241191

EID cannot ensure accessibility for supplementary materials supplied by authors. Readers who have difficulty accessing supplementary content should contact the authors for assistance.

Respiratory Virus Detection and Sequencing from SARS-CoV-2–Negative Rapid Antigen Tests

Appendix 1

Supplementary Methods

Laboratory methods are available at https://dx.doi.org/10.17504/protocols.io.4r3l29pqjv1y/v1

Participants and clinical data

Participants were enrolled in a parent study to evaluate novel viral diagnostic tests as part of the RADx program at the Atlanta Center for Microsystems Engineered Point-of-Care Technologies (ACME-POCT). The study protocol was approved by the Emory Institutional Review Board and the Grady Research Oversight Committee. Participants were enrolled if symptomatic, and they performed testing in a monitored setting to ensure the test was performed correctly. We collected residual negative BinaxNOW test samples from 53 individuals between April-August 2023. Used cassettes containing swabs were stored at 4°C in sealed bags starting from the time of collection. They were transported to the research team in coolers and continued to be stored at 4°C until the time of nucleic acid extraction, which was a median of 6 days after collection (range 2 - 19). Demographic and clinical data were collected in a secure database (REDCap, Nashville, TN). Symptom frequencies were compared between groups (individuals with a virus detected versus those without) using chi-squared tests with Yates correction, or Fisher exact test for comparisons with small numbers.

Nucleic acid extraction and RT-PCR

Nucleic acid extraction was performed using the Qiagen EZ1 DSP Virus Kit with the Advanced XL instrument, per the manufacturer's instructions. RT-PCR was performed to confirm the presence or absence of certain viral targets. A previously published multiplex assay was used for detecting influenza A, influenza B, and SARS-CoV-2 (*1*). RSV RT-PCR was performed using previously published primers (*2*) and the Power SYBR Green RNA-to-CT 1-Step Kit (ThermoFisher Scientific), per the manufacturers' instructions. The samples underwent the following thermocycling conditions: 48°C for 30 minutes, followed by 95°C for 10 minutes, then 45 cycles of 95°C for 15 seconds and 45°C for 45 seconds, with a final melt curve step at 95°C for 15 seconds, 60°C for 15 seconds, and 95°C for 15 seconds.

Metagenomic RNA sequencing

Because RNA extraction used poly-A carrier RNA (cRNA), a depletion step was necessary to remove cRNA before cDNA synthesis. The cRNA depletion was carried out on 5 µL of RNA using a hybridization-based approach as previously described (*3*). Briefly, a 20-mer oligo dT (50 µM, Invitrogen) was added to 5 µL of RNA in a total reaction volume of 10 µL to hybridize to the cRNA. The reaction was incubated at 95°C for 2 minutes, followed by slowly ramping to 45°C at −0.1°C per second. Next, the RNA-DNA hybridization product was treated with 15 units of Hybridase Thermostable RNase H (Biosearch Technologies) and heated for 30 min at 45°C to degrade the poly-A RNA. Next, a DNase treatment step was performed to degrade the residual oligo dT and any DNA in the sample, using the RNase-free DNase kit (Qiagen), according to the manufacturer's instructions. Finally, the cRNA-depleted samples were subjected to a 1.8X bead cleanup using RNAClean XP beads (Beckman Coulter) and eluted in 10 µL of nuclease-free water.

To generate metagenomic libraries for sequencing, double-stranded cDNA was generated from 10 µL of the purified cRNA-depleted samples as follows. Priming was performed with random hexamers at 65°C for 5 minutes, followed by first strand synthesis using the SuperScript IV reverse transcription system (Invitrogen). Second strand synthesis was performed using 10 units of *E. coli* DNA ligase and 40 units of *E. coli* DNA polymerase I (New England Biolabs) in a total reaction volume of 80 µL. This reaction was incubated at 16°C for 2 hours. After incubation, the reaction was stopped by adding EDTA. The resulting double-stranded cDNA was purified using AMPure XP Clean beads (Beckman Coulter) at a 1.8X bead to sample ratio.

Illumina-compatible libraries were prepared from 4 µL of the purified double-stranded cDNA using the Nextera XT kit (Illumina) following the manufacturer's recommendations. The resulting unique, dual-indexed libraries were subjected to a 0.7X bead cleanup step using AMPure XP beads (Beckman Coulter). The purified libraries were quantified using the KAPA quantification kit (Roche), normalized, and pooled. The resulting pool of samples was then cleaned a final time using 0.8X DNA AMPure XP beads (Beckman Coulter), and the pool was quantified using the KAPA quantification kit (Roche), diluted to a loading concentration of 10 pM, and sequenced using an Illumina MiSeq or NextSeq 2000. A median of 5.8 million reads per sample was obtained (range 0.6–40.2 million, Appendix 2 Table). Ten water samples were included as negative controls. As a positive control, 1 pg of unique synthetic RNA fragments (ERCC, Jiang et al 2011) were added to each sample during the cRNA depletion step.

Metagenomic analysis

We used a three-step bioinformatic approach to detect and confirm viruses in each sample (Appendix 1 Figure 1). Reads first underwent deduplication using clumpify v39.06, quality trimming and adaptor removal using Trimmomatic v0.39 (4-base wide sliding window, minimum average quality of 15, leading and trailing base removal quality below 3), and further quality filtering and merging of paired reads using Fastp v0.23.2 (default settings, minimum length 50). Filtered reads underwent metagenomic classification with KrakenUniq v0.5.7, and viral reads were confirmed with blastn v2.12.0 against the BLAST nucleotide database Version 4. A custom python script used the NCBI taxonomy utility (updated 4/22/24) to determine the lowest common ancestor (LCA) between the top three results from KrakenUniq and blastn; if no viral LCA was identified, the read was designated a false-positive result from KrakenUniq.

For viral taxa confirmed by both KrakenUniq and blastn, the closest matching reference sequence in GenBank was identified using a custom script, as follows. This script, requiring the NCBI Taxonomy ID (taxID), first downloads genomic data from NCBI using the datasets download genome taxon command, saving it as tax_$taxID.zip and extracting it into dir_$taxID. It then concatenates all FASTA files in the extracted directory into a single file, all_$taxID.fna, by generating a list of .fna files and appending their contents. Finally, the script uses the makeblastdb command to create a nucleotide BLAST database named candidate_virus from the consolidated FASTA file. This pipeline ensures efficient genomic data processing and BLAST database preparation for subsequent bioinformatics analyses.

The next script in the pipeline processes zipped paired-end reads (`R1` and `R2`), concatenates them into a single FASTQ file, and converts this into a FASTA file. It then filters the sequences based on a provided CSV file, extracting only the blast-verified reads. The resulting subset of sequences is saved in a new FASTA file for further analysis.

The final script in the pipeline processes input files to identify and analyze sequence data against a BLAST database. It begins by running a BLAST search on the input FASTA file (`ffn`) against the specified BLAST database (`blastDB`), outputting the results in tabular format. The script identifies the reference sequence with the highest number of positive blast hits above an 80% identity threshold and generates a BED file listing the aligned regions. It then subsets the original FASTQ file to include only reads aligning to this reference sequence. The script calculates the percent coverage of the reference sequence by comparing the lengths of aligned regions and the reference sequence itself. Finally, it generates an output file summarizing the read count, reference sequence, taxonomy ID, region count, aligned region length, reference length, and percent coverage. This automated pipeline facilitates the detailed analysis of sequence data, providing insights into genomic alignments and coverage.

Appendix 3 lists all taxa identified by KrakenUniq and blastn within each sample and presents the results of reference mapping. In some cases, a virus was reported by both KrakenUniq and blastn, however no reads were confirmed by reference mapping; these results are specified in Appendix 3 Table 3, and these taxa are included in the plots shown in Appendix 1 Figure 3 (light blue squares) but not Figure 3. In other cases, a small number of viral reads were confirmed by reference mapping, but not enough to confidently report the virus as present in the sample; these results are specified in Appendix 3 Table 2 and are included in the plots shown in both Appendix 1 Figure 3 and Figure 3 (light blue squares). A virus was only confirmed as present in a sample if there was at least 10% genome coverage or there were reads mapping to at least 3 nonoverlapping regions of the genome, a threshold based on clinical diagnostic studies using metagenomic sequencing (*4*). Viruses that were found in multiple study samples and negative controls were regarded as environmental or reagent contaminants, including mastadenovirus C, which was found at low levels (<5% genome coverage, with reads only detected in the middle third of the genome) in 20 samples (38%) and 3 controls (30%).

Phylogenetic analysis

For all confirmed viruses, reference-based assembly was performed using viralrecon v2.6.0 and the reference sequences listed in Appendix 2.

Phylogenetic analysis was performed for parainfluenza 3: reference sequences were obtained from GenBank (N = 451) using the name “human respirovirus 3” (taxid 11216) and following conditions: sequence length 14,000–16,000 nt, complete genomes, and human hosts (taxid 9606). Identical sequences were removed, leading to a final alignment of 423 reference sequences. The four parainfluenza 3 sequences obtained in this study were aligned with the 423 references and NC_075446.1 using MAFFT v7.505. A maximum likelihood phylogenetic tree was constructed with IQ-TREE v2.3.5, with the GTR+F+I+R5 model and 1000 ultrafast bootstrap replicates. For visualization purposes, the tree was downsampled using UShER to generate subtrees with reference sequences closely related to the sequences from this study. All trees were visualized and annotated with iTOL v6.9.1.

References

1. Shu B, Kirby MK, Davis WG, Warnes C, Liddell J, Liu J, et al. Multiplex real-time reverse transcription PCR for influenza A virus, influenza B virus, and severe acute respiratory syndrome coronavirus 2. Emerg Infect Dis. 2021;27:1821–30. PubMed https://doi.org/10.3201/eid2707.210462

2. Todd AK, Costa AM, Waller G, Daley AJ, Barr IG, Deng YM. Rapid detection of human respiratory syncytial virus A and B by duplex real-time RT-PCR. J Virol Methods. 2021;294:114171. PubMed https://doi.org/10.1016/j.jviromet.2021.114171

3. Matranga CB, Gladden-Young A, Qu J, Winnicki S, Nosamiefan D, Levin JZ, et al. Unbiased Deep Sequencing of RNA Viruses from Clinical Samples. J Vis Exp. 2016;113:54117. **PMID 27403729**

4. Miller S, Naccache SN, Samayoa E, Messacar K, Arevalo S, Federman S, et al. Laboratory validation of a clinical metagenomic sequencing assay for pathogen detection in cerebrospinal fluid. Genome Res. 2019;29:831–42. PubMed https://doi.org/10.1101/gr.238170.118

Appendix 1 Table. Participant demographics

Characteristic	Total Participants = 53
Sex	
M	17 (32.1%)
F	36 (66.1%)
Race	
Black or African American	37 (69.8%)
White	7 (13.2%)
Asian	2 (3.7%)
Native Hawaiian or Other Pacific Islander	0 (0.0%)
American Indian / Alaskan Native	0 (0.0%)
Other	6 (11.3%)
Unknown	1 (1.9%)
Ethnicity	
Hispanic or Latino	4 (7.6%)
Not Hispanic or Latino	49 (92.5%)
Age Range	
20–29	10 (18.9%)
30–39	13 (24.5%)
40–49	8 (15.1%)
50–59	7 (13.2%)
60–69	10 (18.9%)
70–79	4 (7.6%)
80–89	1 (1.9%)

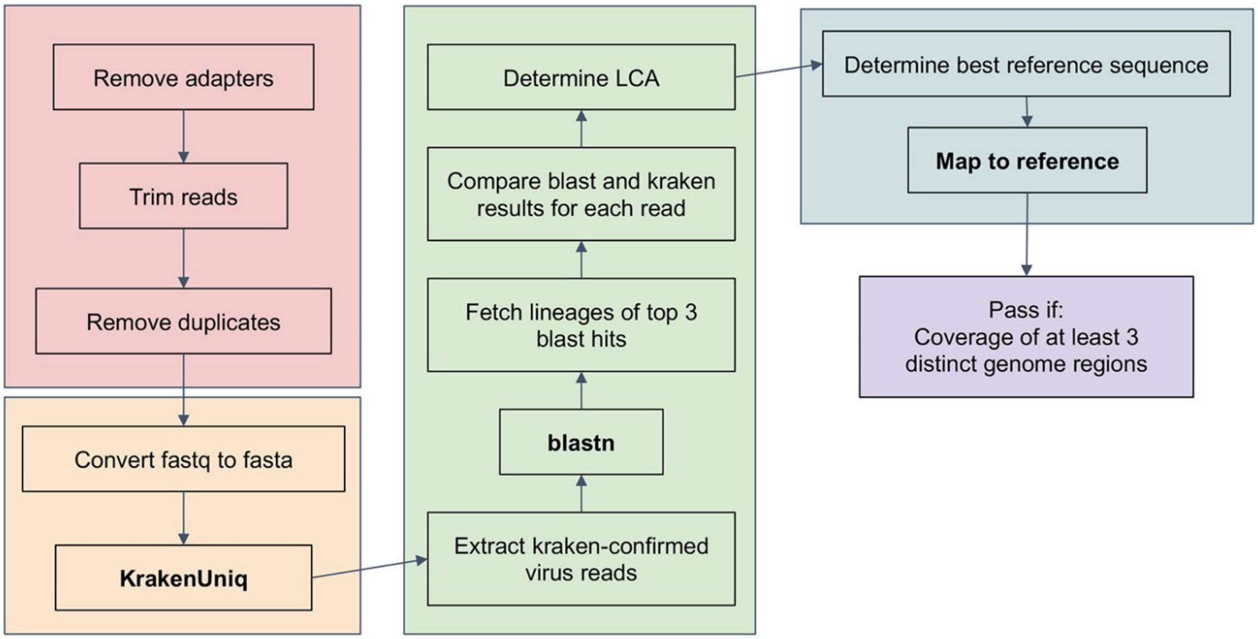

Appendix 1 Figure 1. Analysis pipeline for metagenomic classification and confirmation. Red boxes describe processing and quality control steps, yellow boxes describe initial metagenomic classification using KrakenUniq, green boxes describe blastn confirmation of reads classified as viral by KrakenUniq, and blue boxes describe reference-based mapping for final confirmation. LCA = lowest common ancestor.

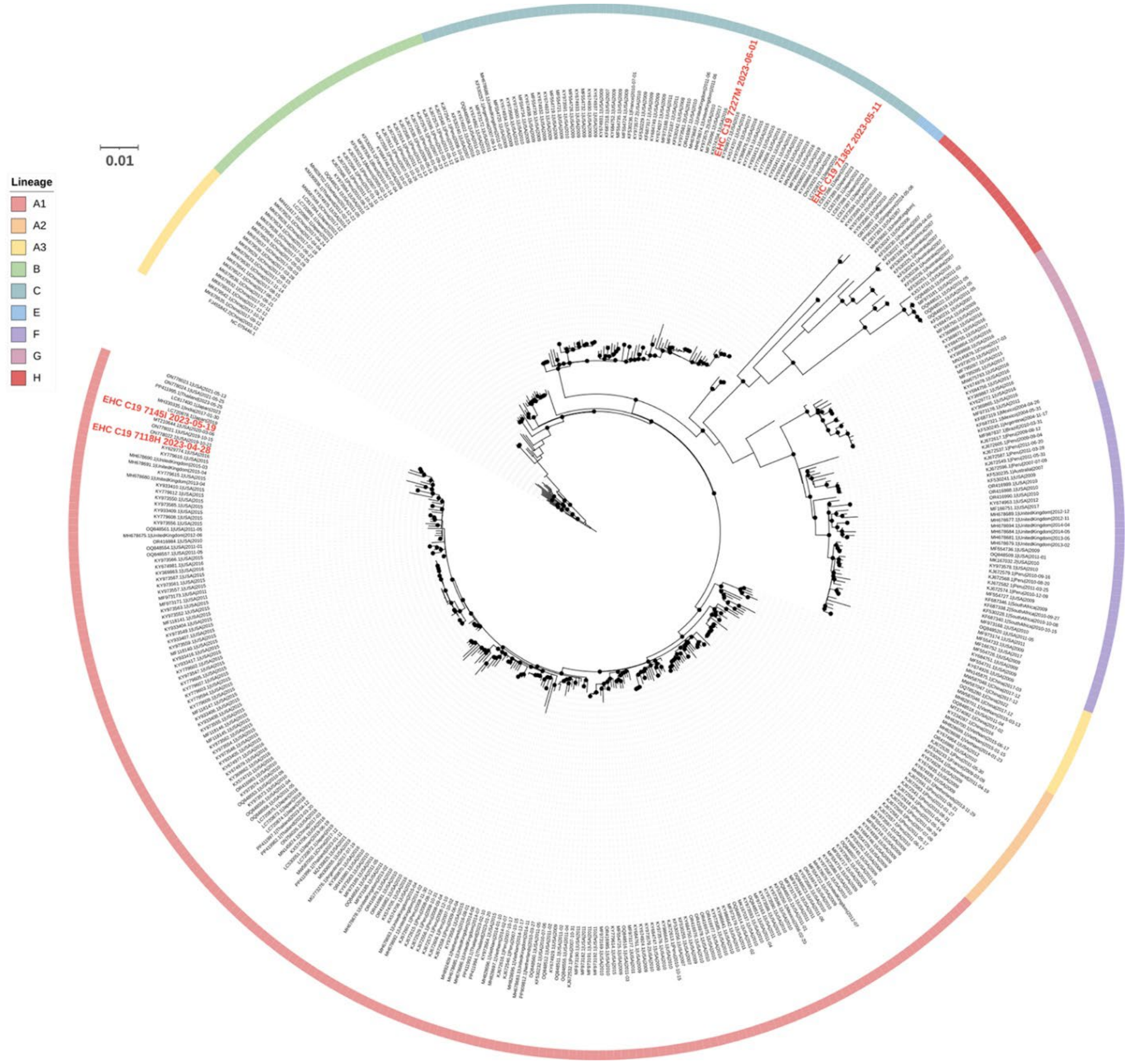

Appendix 1 Figure 2. Maximum likelihood phylogenetic analysis of parainfluenza 3 virus sequences. The names of sequences obtained in this study are bold and in red, and reference sequences in black represent all unique full-length genome sequences of parainfluenza 3 available in GenBank (7/30/24). Circles indicate nodes with >95% ultrafast bootstrap support. The outer ring indicates virus lineage.

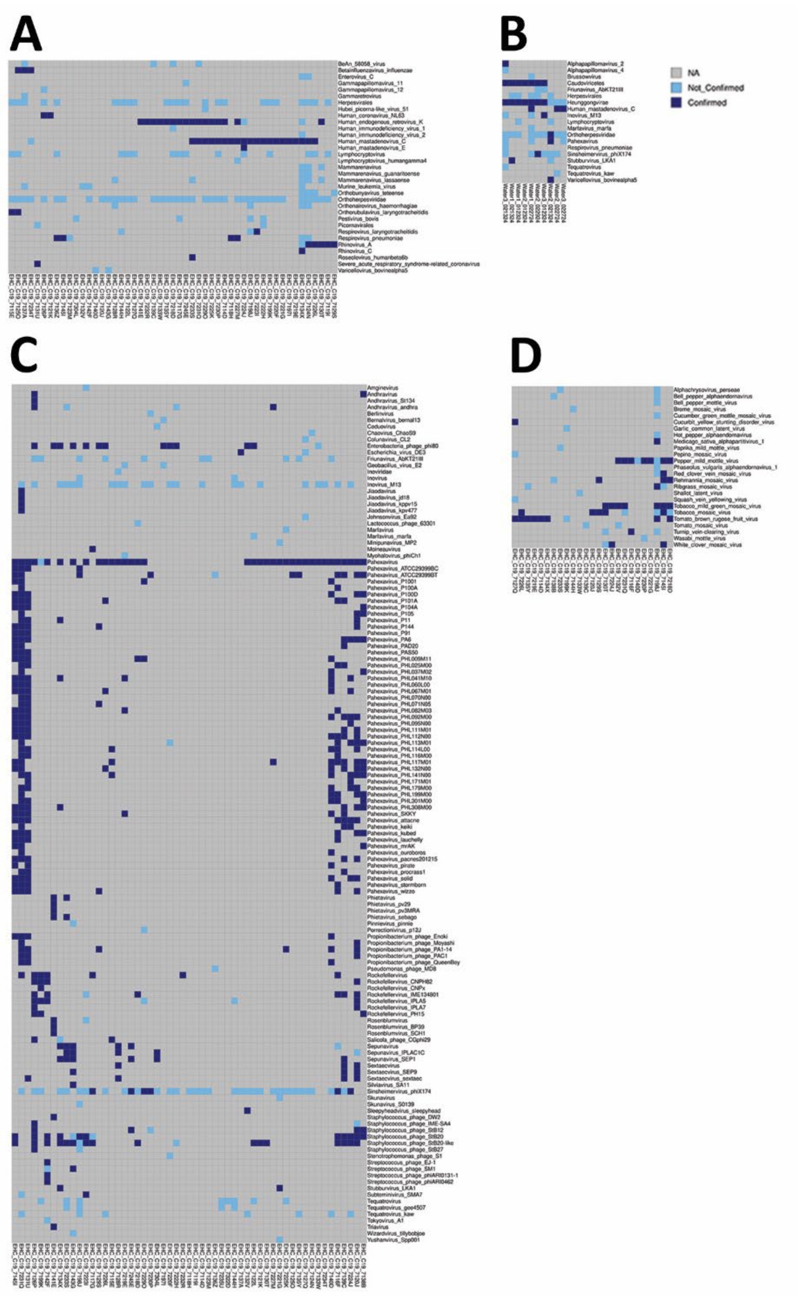

Appendix 1 Figure 3. Plot of the viral taxa (rows) that were detected in each sample (columns). Light blue boxes indicate viruses that were detected by both KrakenUniq and blastn but not confirmed by reference mapping, while dark blue boxes indicate viruses that were detected by both KrakenUniq and blastn and were confirmed by reference mapping (covering at least 3 distinct regions or 10% of the reference virus genome). Unlike Figure 3, the light blue boxes in this figure include viruses that were reported by both KrakenUniq and blastn but had zero reads confirmed by reference mapping. For ease of visualization, panel **A)** shows animal viruses detected in samples; panel **B)** shows all viruses detected in negative controls; panel **C)** shows bacteriophages detected in samples; and panel **D)** shows plant viruses detected in samples.

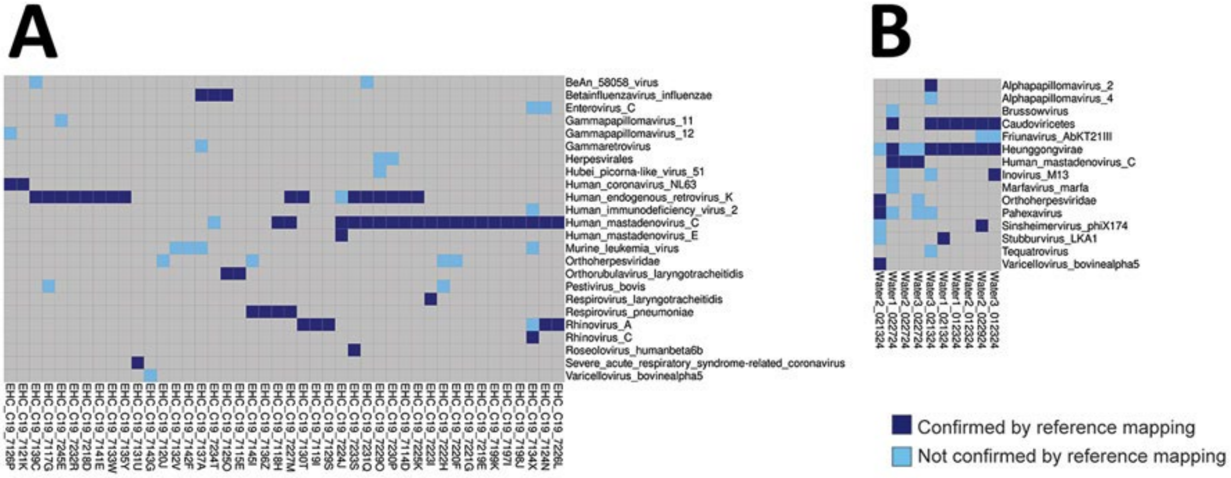

Appendix 1 Figure 4. Plot of the viral taxa (rows) that were detected in each sample (columns). Panel A) shows results from samples in this study, while panel B) shows results from negative controls. Dark blue boxes indicate viruses that were detected by both KrakenUniq and blastn and were confirmed by reference mapping (covering at least 3 distinct regions or 10% of the reference virus genome). Light blue boxes indicate viruses that were detected by both KrakenUniq and blastn but not confirmed by reference mapping. This figure only includes results for which at least one read mapped to a reference genome sequence. Appendix 1 Figure 3 panels A) and B) include viruses that were reported by KrakenUniq and blastn, but had zero reads mapping to a reference virus genome. Bacteriophages and plant-infecting viruses are shown in Appendix 1 Figure 3 panels C) and D), respectively.
